# Author Correction: High-throughput and high-efficiency sample preparation for single-cell proteomics using a nested nanowell chip

**DOI:** 10.1038/s41467-021-27110-0

**Published:** 2021-11-30

**Authors:** Jongmin Woo, Sarah M. Williams, Lye Meng Markillie, Song Feng, Chia-Feng Tsai, Victor Aguilera-Vazquez, Ryan L. Sontag, Ronald J. Moore, Dehong Hu, Hardeep S. Mehta, Joshua Cantlon-Bruce, Tao Liu, Joshua N. Adkins, Richard D. Smith, Geremy C. Clair, Ljiljana Pasa-Tolic, Ying Zhu

**Affiliations:** 1 Environmental Molecular Sciences Laboratory, Pacific Northwest National Laboratory, Richland, WA 99354 USA; 2 Biological Sciences Division, Pacific Northwest National Laboratory, Richland, WA 99354 USA; 3 Scienion AG, Volmerstraße 7, 12489 Berlin, Germany; 4 Cellenion SASU, 60 Avenue Rockefeller, Bâtiment BioSerra2, 69008 Lyon, France

**Keywords:** Lab-on-a-chip, Mass spectrometry, Proteomics, Proteome

Correction to: *Nature Communications* 10.1038/s41467-021-26514-2, published online 29 October 2021.

In this article the author name Chia-Feng Tsai was incorrectly written as Chai-Feng Tsai.

The grant number U01 HL148860 relating to NIH grants for Joshua N. Adkins and Geremy C. Clair was omitted. The original article has been corrected.

