# Supplementary material for: High-throughput and high-efficiency sample preparation for single-cell proteomics using a nested nanowell chip
Source: Nat Commun. 2021 Oct 29;12:6246. doi: 10.1038/s41467-021-26514-2 (PMC8556371; doi:10.1038/s41467-021-26514-2)
Supplement: Supplementary file 1 — Supplementary Information [file 41467_2021_26514_MOESM1_ESM.pdf]

Supplementary Information for

High-throughput and high-efficiency sample preparation for single-cell proteomics using a nested nanowell chip

Jongmin Woo,[1] Sarah M. Williams,[1] Lye Meng Markillie,[1] Song Feng,[2] Chai-Feng Tsai,[2] Victor Aguilera-Vazquez,[1] Ryan L. Sontag,[2] Ronald J. Moore,[2] Dehong Hu,[1] Hardeep S. Mehta,[1] Joshua Cantlon-Bruce,[3,4] Tao Liu,[2] Joshua N. Adkins,[2] Richard D. Smith,[2] Geremy C. Clair,[2] Ljiljana Pasa-Tolic,[1] Ying Zhu[1,]*

[1]Environmental Molecular Sciences Laboratory, Pacific Northwest National Laboratory, Richland, Washington 99354, United States

[2]Biological Sciences Division, Pacific Northwest National Laboratory, Richland, Washington 99354, United States

[3]Scienion AG, Volmerstraße 7, 12489 Berlin, Germany

[4]Cellenion SASU, 60 Avenue Rockefeller, Bâtiment BioSerra2, 69008 Lyon, France

***Corresponding author:**

Dr. Ying Zhu (ying.zhu@pnnl.gov)

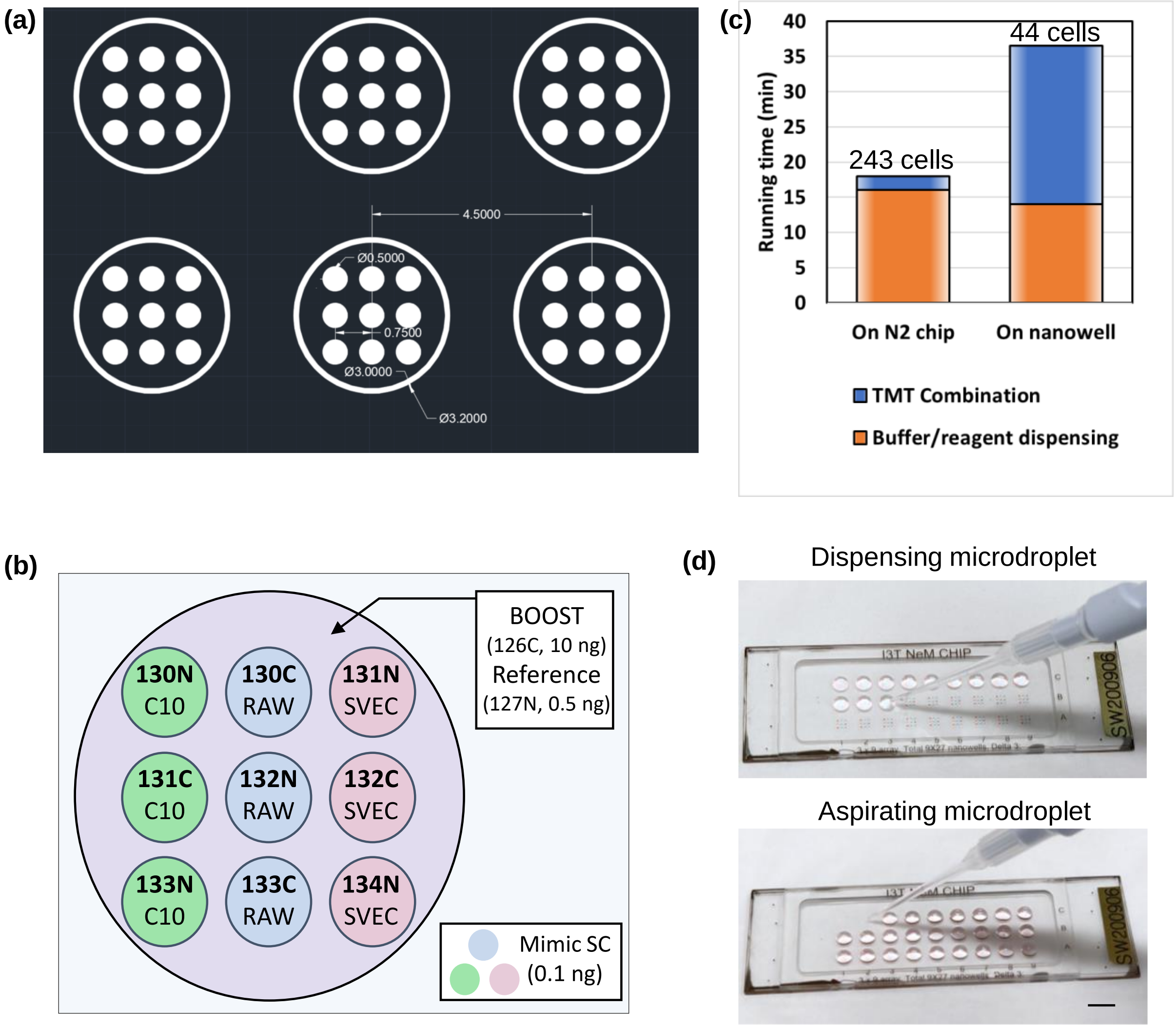

Supplementary figure 1. Design and operation of N2 chip. (a) Schematic illustration showing the N2 chip design. The white areas are designed for nanowells and hydrophilic rings, while the rest is hydrophobic surface. (b) Experiment design for mimic single-cell sample (0.1 ng peptide) on the N2 chip. (c) Estimated robot operation time for single cell proteomics using N2 chip and nanowell chip. (d) Photographs showing TMT-based samples can be pooled together by spotting a 8-µL droplet using a micropipette. Similarly, the pooled sample can be retrieved and loaded into an autosampler vial for LC-MS analysis. The length of scale bar is 5 mm.

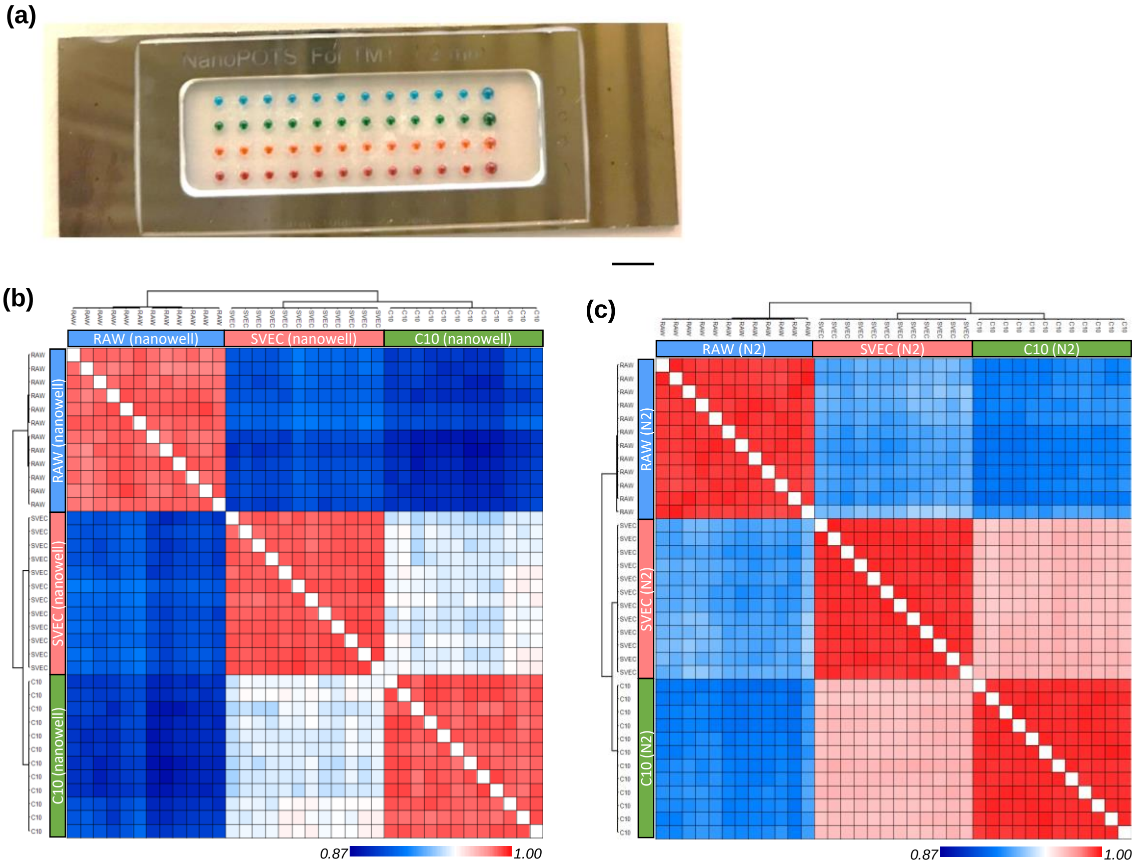

Supplementary figure 2. Performance comparison between previous nanowell chip and N2 chip. (a) A photo of original nanowell chip. The length of scale bar is 5 mm. (b,c) Heatmap of pairwise Pearson correlations among individual samples i n nanowell chip (b) and N2 chip (c). Source data are provided.

(a)

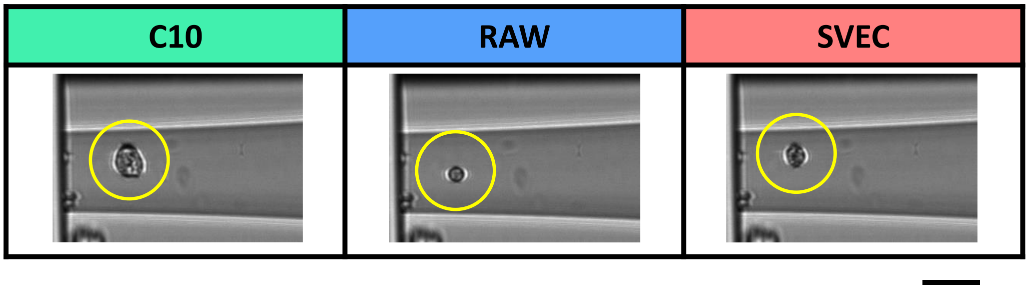

(b)

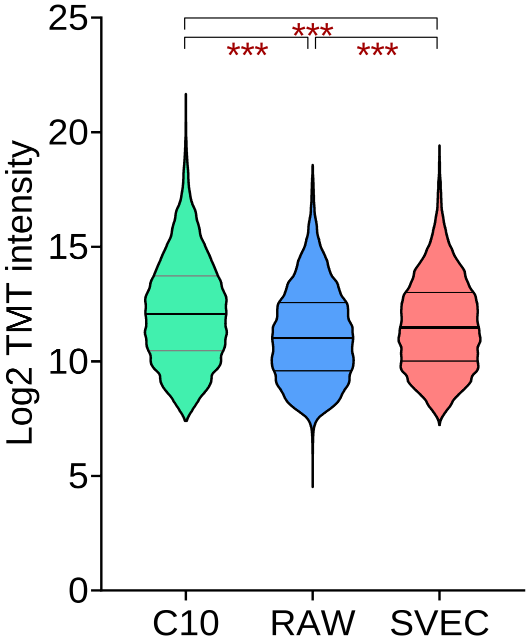

Supplementary figure 3. The correlations of cell sizes and protein intensities. (a) Representative images of single cells. The measured cell sizes in diameter are 18-20 µm for C10 cells, 7-10 µm for RAW cells, and 13-15 µm for SVEC cells. The length of scale bar is 50 µm. (b) Violin plots showing the distribution of log2 transformed protein intensities for the three cell types (n=20854 for C10; n=20020 for RAW; n=20884 for SVEC. Center lines show the medians; top and bottom horizontal lines indicate the 25th and 75th percentiles, respectively. A two-sided t-test was applied to confirm the significance between different cell types (*p-value* ***<0.001). Source data are provided.

(a)

TMT	Cell tye	Input (ng)
126	MOLM-14	0.1
127N	K562	0.1
127C	CMK	0.1
128N	MOLM-14	0.1
128C	K562	0.1
129N	MOLM-14	0.1
129C	CMK	0.1
130N	Empty	0
130C	MOLM-14	0.1
131	Mix	10

TMTpro	Cell tye	Input (ng)
126	MOLM-14	0.1
127N	K562	0.1
127C	CMK	0.1
128N	MOLM-14	0.1
128C	K562	0.1
129N	CMK	0.1
129C	MOLM-14	0.1
130N	K562	0.1
130C	CMK	0.1
131N	MOLM-14	0.1
131C	K562	0.1
132N	CMK	0.1
132C	MOLM-14	0.1
133N	Empty	0
133C	CMK	0.1
134N	Mix	10

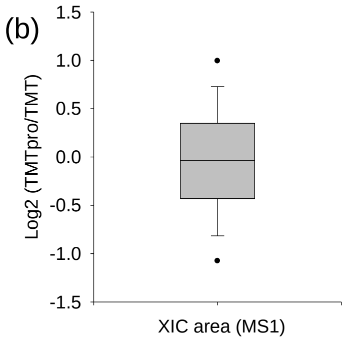

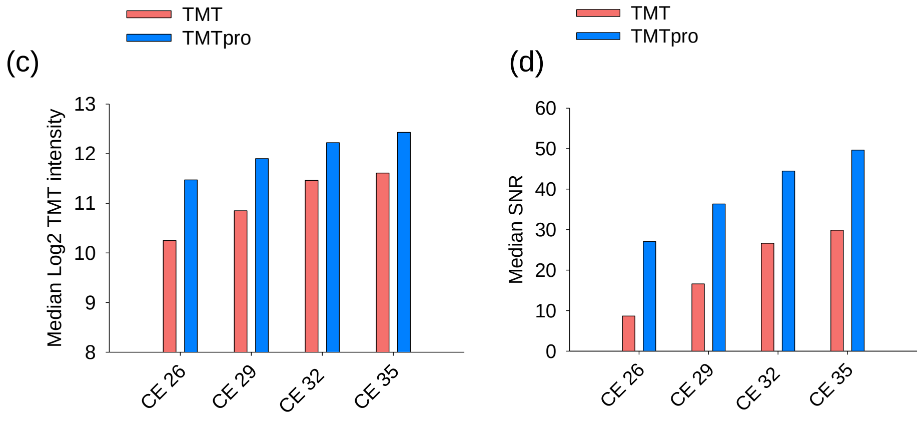

Supplementary figure 4. Comparison of the signal intensities and SNRs between TMT 10plex and TMTpro 16plex-labeled peptides. (a) Experimental designs. (b) The MS 1 signal difference between the two TMT reagents (n = 7216 ratio values used to draw the box plot). Centerline shows the median; box limits indicate the 25th and 75th percentiles; whiskers extend 1.5 times the interquartile range from the 25th and 75th percentiles. (c) The comparison of median log2-transformed reporter ion intensities and (d) median SNRs per channel at different normalized HCD collision energies. Source data are provided.

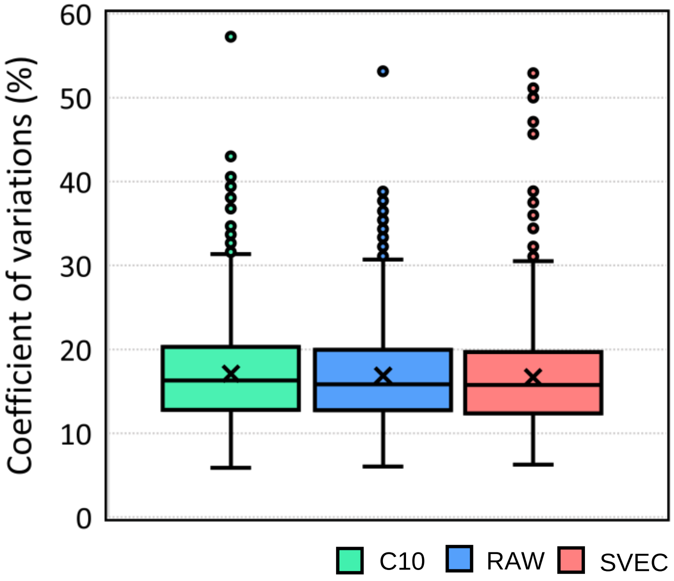

Supplementary figure 5. The distribution of coefficient of variations (CVs) for protein abundances in single cells among inter TMT batches with batch correction. For each cell type, 36 single cells from 12 TMT sets were used for the calculation. Center lines show the medians; cross show the means; box limits indicate the 25th and 75th percentiles; whiskers extend 1.5 times the interquartile range from the 25th and 75th percentiles. Source data are provided.

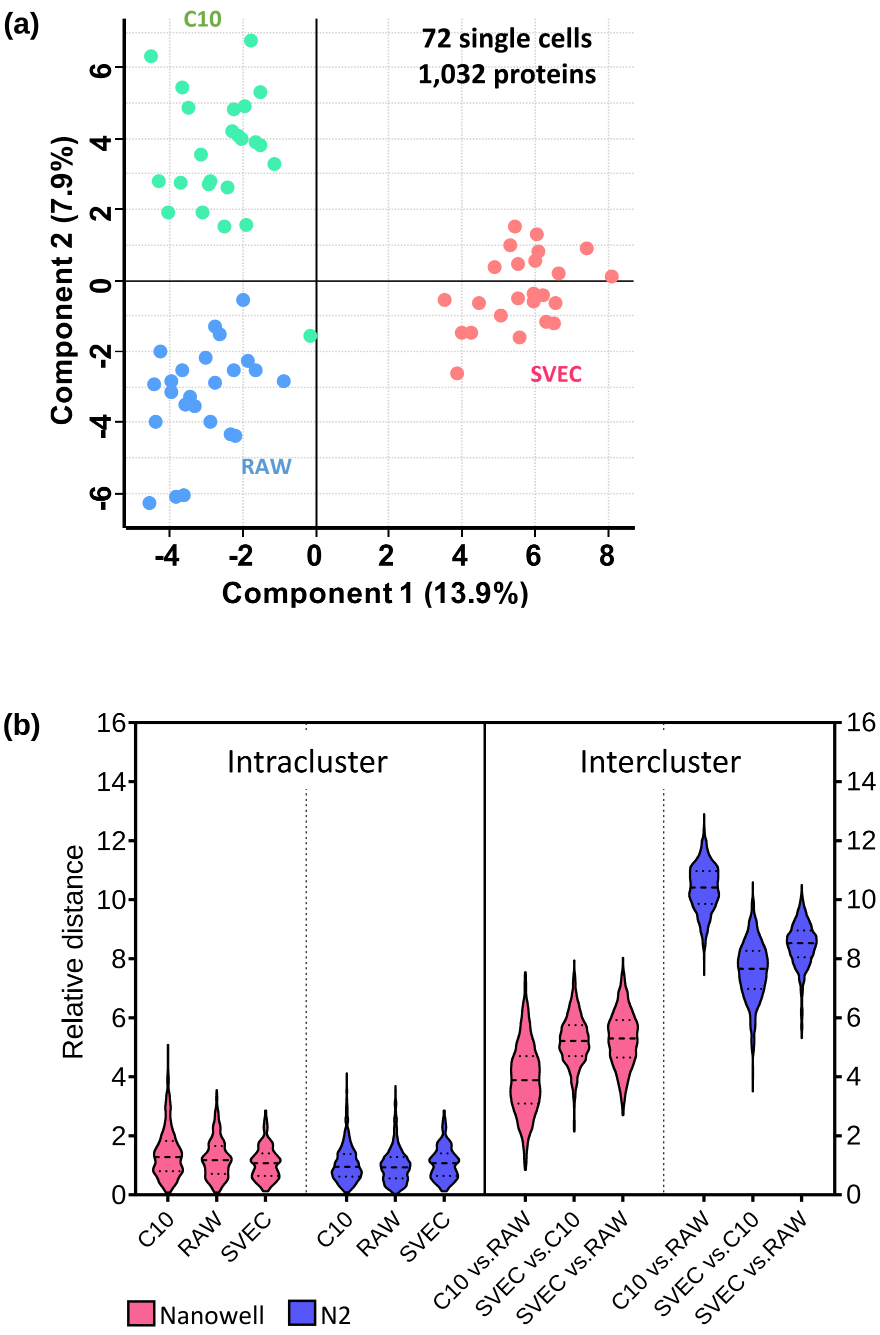

Supplementary figure 6. The comparison of cell clustering performance between previous nanowell chip and N2 chip. (a) PCA plot showing the clustering of 72 single cells using nanowell chips[1]. Total 1,032 proteins were used. (b) A cluster distance analysis for PCA clusters in nanowell or N2 methods. Intracluster distance is the distance between two objects belonging to same cluster, while intercluster distance is the distance between two objects belonging to two different clusters. Center lines show the medians; top and bottom horizontal lines indicate the 25th and 75th percentiles, respectively. In the intracluster plot, the numbers of distance values (n) from nanowell and N2 datasets are 276 and 630, respectively; In the intercluster plot, the number of the distance values (n) from nanowell and N2 datasets are 576 and 1296, respectively. Source data are provided.

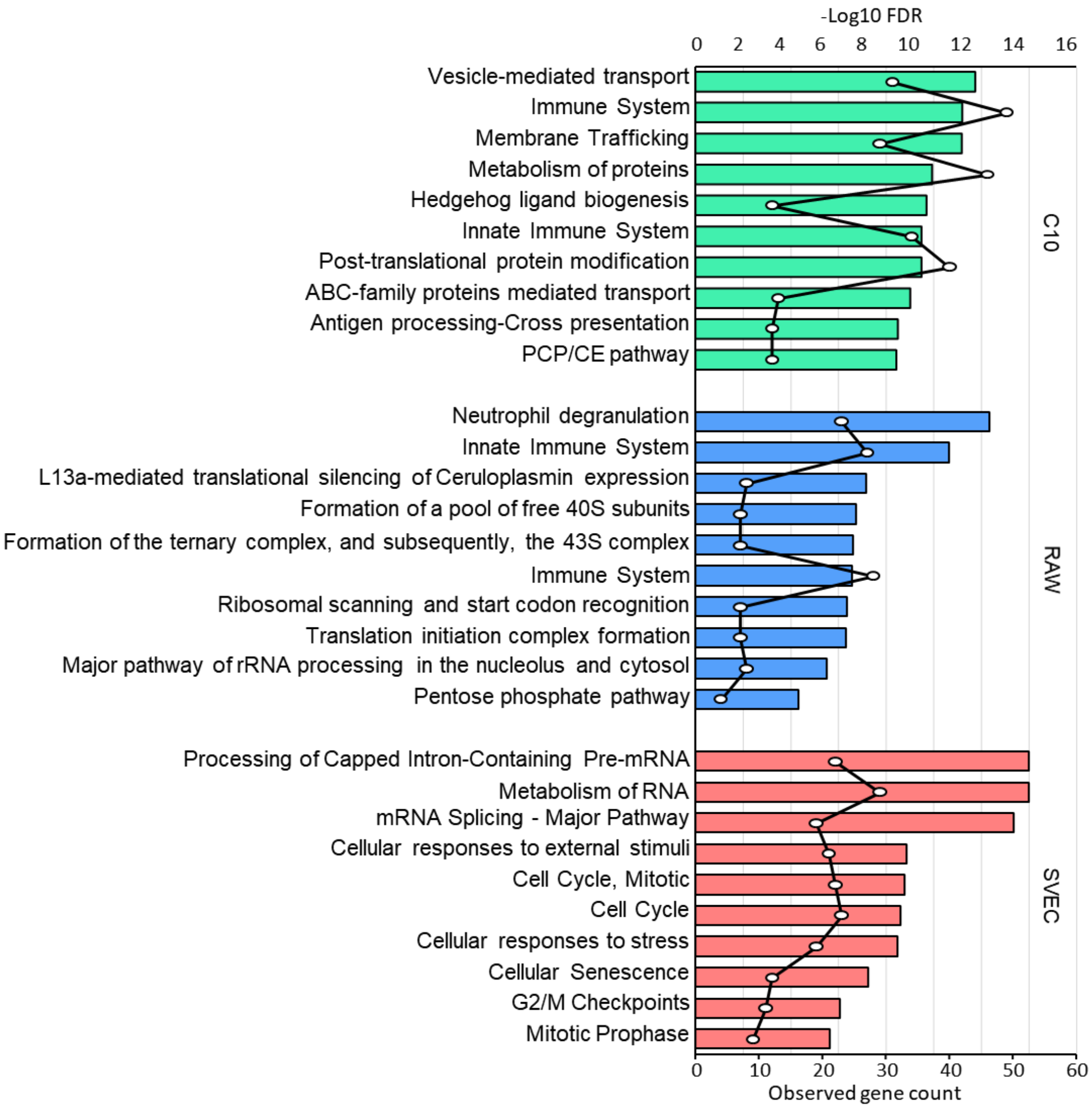

Supplementary figure 7. Reactome pathway analysis of enriched proteins in each cell type by hierarchical clustering analysis. The top 10 of the pathways per each homogeneous cell type based on adjusted *p-value* were listed with the number of observed protein counts. Source data are provided.

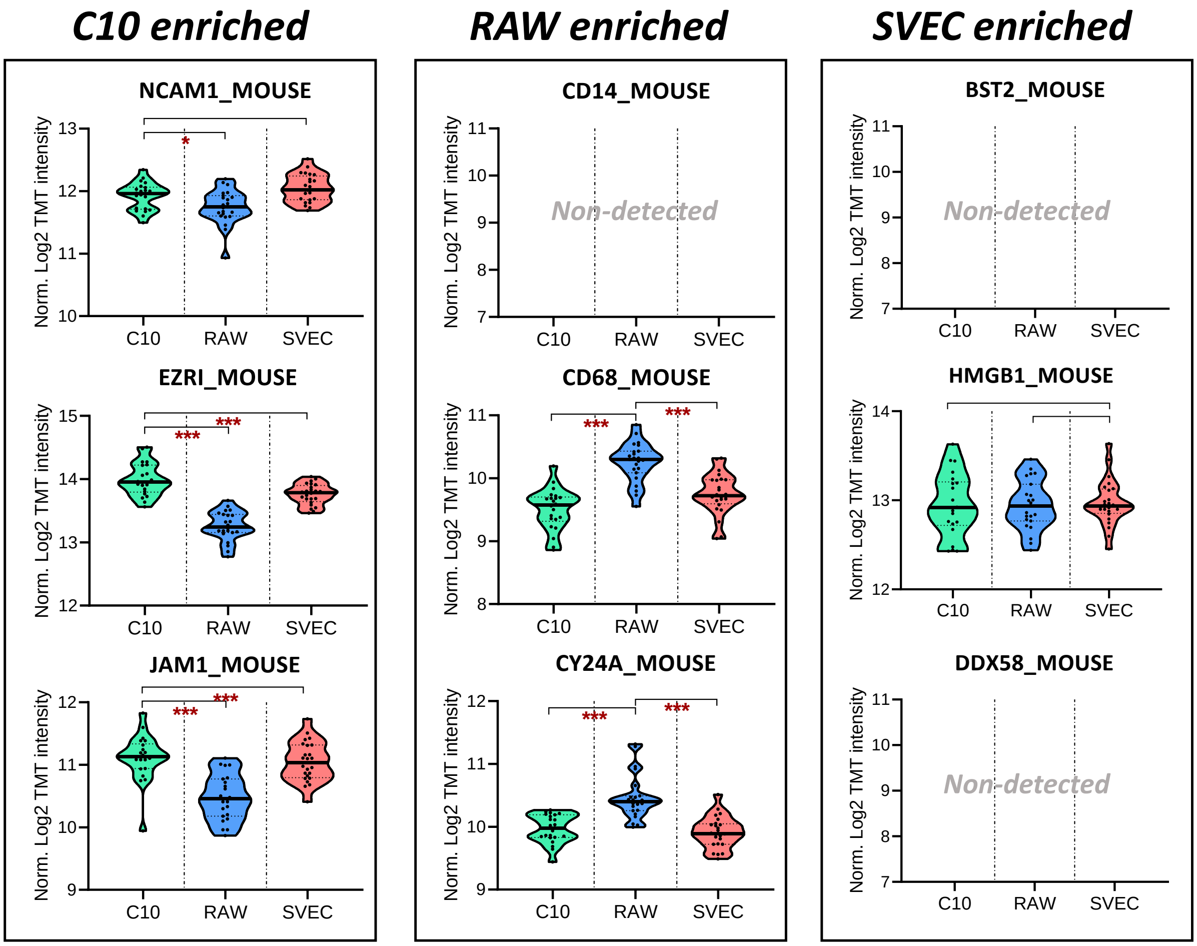

Supplementary figure 8. Violin plots showing the intensity distributions of putative plasma membrane protein markers for specific cell types using previous nanowell-chip-based single-cell proteomics data.[1] The protein lists were selected based on the data from the N2 chips and shown here to compare the improved proteome coverage and quantitation performance of the N2 chip. The significances were analyzed using a two-sided t-test (*p-value* *<0.05, **<0.01, ***<0.001). Center lines show the medians; top and bottom horizontal lines indicate the 25th and 75th percentiles, respectively. For C10, n=22 single cells; For RAW, n = 23 single cells; For SVEC, n = 24 single cells. Source data are provided.

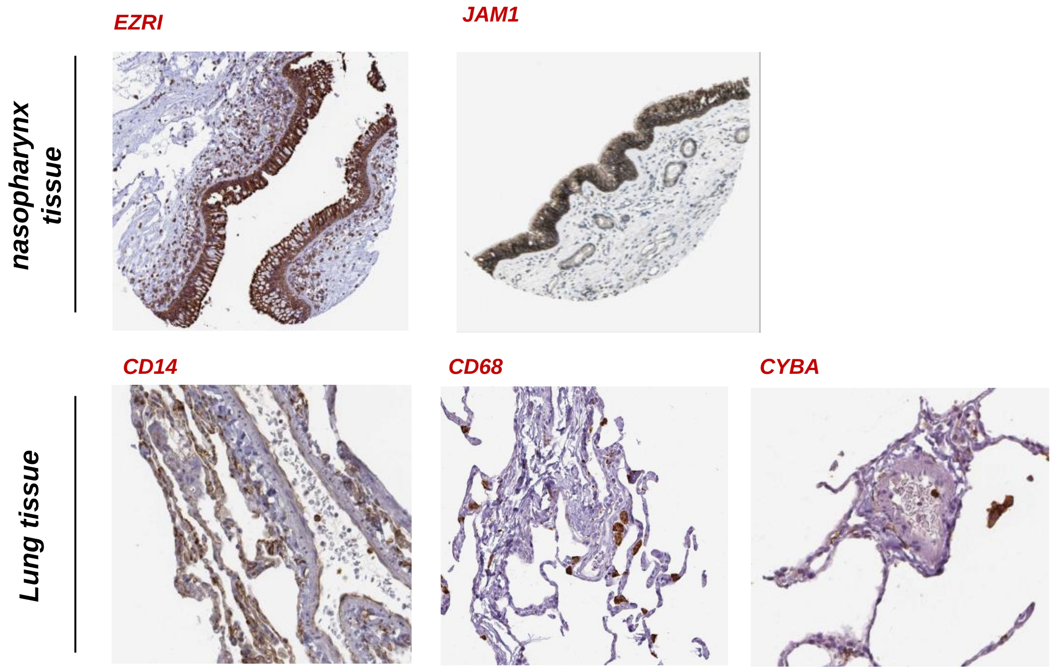

Supplementary figure 9. Immunohistochemistry staining images of highly enriched membrane protein makers on specific cell types. Upper images show EZRI and JAM1 enriched in C10 cells are localized in epithelial cells of human nasopharynx tissues; Bottom images show CD14, CD68, and CYBA (Uniprot name: CY24A_Human) enriched in RAW cells specifically localized in macrophase cells of human lung tissue. All the images were downloaded from human protein atlas database (https://www.proteinatlas.org/).

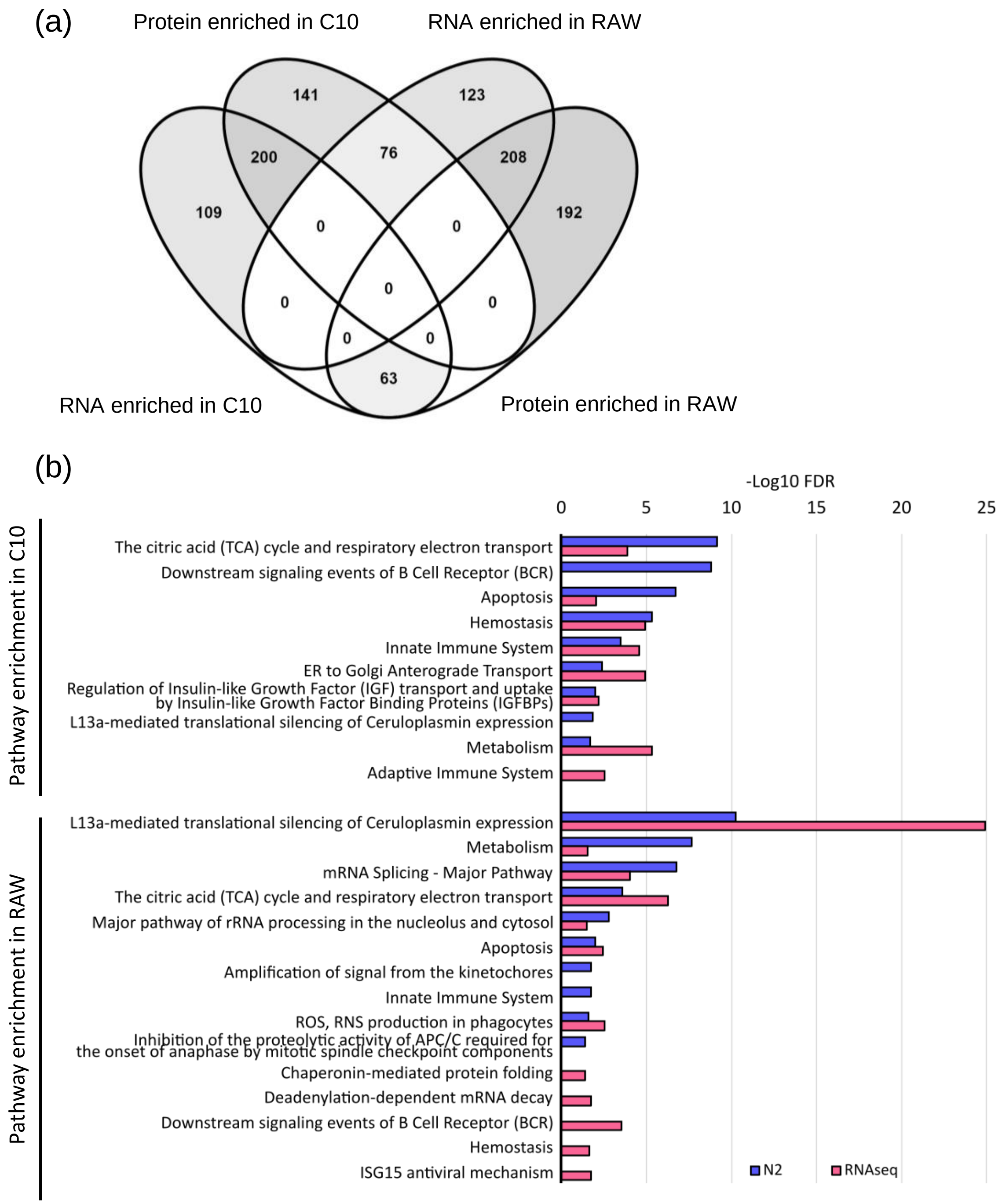

Supplementary figure 10. Enrichment analysis based on scProteomics and scRNA-seq. (a) Venn diagram showing the overlaps of enriched mRNA and proteins in the two cell types. (b) . Reactome pathway analysis of enriched mRNA and proteins in the two cell types. A two-sided t-test with *p-value* of <0.05 was used to generate the significantly enriched targets. Source data are provided.

Supplementary references.

1. Dou, M. et al. High-Throughput Single Cell Proteomics Enabled by Multiplex Isobaric Labeling in a Nanodroplet Sample Preparation Platform. *Anal Chem* **91**, 13119-13127 (2019).
