# Supplementary material for: High-throughput and high-efficiency sample preparation for single-cell proteomics using a nested nanowell chip
Source: Nat Commun. 2021 Oct 29;12:6246. doi: 10.1038/s41467-021-26514-2 (PMC8556371; doi:10.1038/s41467-021-26514-2)
Supplement: Supplementary file 3 — Description of Additional Supplementary Files [file 41467_2021_26514_MOESM3_ESM.docx]

**Description of additional supplementary files for**

**High-throughput and high-efficiency sample preparation for single-cell proteomics using a nested nanowell chip**

Jongmin Woo,^1^ Sarah M. Williams,^1^ Lye Meng Markillie,^1^ Song Feng,^2^ Chai-Feng Tsai,^2^ Victor Aguilera-Vazquez,^1^ Ryan L. Sontag,^2^ Ronald J. Moore,^2^ Dehong Hu,^1^ Hardeep S. Mehta,^1^ Joshua Cantlon-Bruce,^3,4^ Tao Liu,^2^ Joshua N. Adkins,^2^ Richard D. Smith,^2^ Geremy C. Clair,^2^ Ljiljana Pasa-Tolic,^1^ Ying Zhu^1,^*

^1^Environmental Molecular Sciences Laboratory, Pacific Northwest National Laboratory, Richland, Washington 99354, United States

^2^Biological Sciences Division, Pacific Northwest National Laboratory, Richland, Washington 99354, United States

^3^Scienion AG, Volmerstraße 7, 12489 Berlin, Germany

^4^Cellenion SASU, 60 Avenue Rockefeller, Bâtiment BioSerra2, 69008 Lyon, France

***Corresponding author:**

Dr. Ying Zhu ([ying.zhu@pnnl.gov](mailto:ying.zhu@pnnl.gov))

File Name: Supplementary Data 1

Description: SCPCompanion output tables from this study and datasets of previous studies (Dou et al., 2018, Analytical Chemistry; Tsai et al. 2020, Molecular Cellular Proteomics).

File Name: Supplementary Data 2

Description: Surface marker proteins predicted by scProteomics, scRNA-seq, and the overlap of scProteomics and scRNA-seq. Proteins labeled with red were validated by immunofluorescence imaging.

File Name: Supplementary Data 3

Description: Single-cell RNA sequencing data for C10 cells obtained from a previous study (Mitchell, et al., 2016, Acs Nano)
