# Supplementary material for: High-throughput and high-efficiency sample preparation for single-cell proteomics using a nested nanowell chip
Source: Nat Commun. 2021 Oct 29;12:6246. doi: 10.1038/s41467-021-26514-2 (PMC8556371; doi:10.1038/s41467-021-26514-2)
Supplement: Supplementary file 7 — Reporting Summary [file 41467_2021_26514_MOESM7_ESM.pdf]

nature portfolio

Corresponding author(s): Dr.Ying Zhu

Last updated by author(s): Sep 16, 2021

Reporting Summary

Nature Portfolio wishes to improve the reproducibility of the work that we publish. This form provides structure for consistency and transparency in reporting. For further information on Nature Portfolio policies, see our Editorial Policies and the Editorial Policy Checklist.

Statistics

For all statistical analyses, confirm that the following items are present in the figure legend, table legend, main text, or Methods section.

n/a	Confirmed	
☐	☒	The exact sample size (n) for each experimental group/condition, given as a discrete number and unit of measurement
☐	☒	A statement on whether measurements were taken from distinct samples or whether the same sample was measured repeatedly
☐	☒	The statistical test(s) used AND whether they are one- or two-sided<br>*Only common tests should be described solely by name; describe more complex techniques in the Methods section.*
☐	☒	A description of all covariates tested
☐	☒	A description of any assumptions or corrections, such as tests of normality and adjustment for multiple comparisons
☐	☒	A full description of the statistical parameters including central tendency (e.g. means) or other basic estimates (e.g. regression coefficient) AND variation (e.g. standard deviation) or associated estimates of uncertainty (e.g. confidence intervals)
☒	☐	For null hypothesis testing, the test statistic (e.g. F, t, r) with confidence intervals, effect sizes, degrees of freedom and P value noted<br>*Give P values as exact values whenever suitable.*
☒	☐	For Bayesian analysis, information on the choice of priors and Markov chain Monte Carlo settings
☐	☒	For hierarchical and complex designs, identification of the appropriate level for tests and full reporting of outcomes
☐	☒	Estimates of effect sizes (e.g. Cohen's d, Pearson's r), indicating how they were calculated

Our web collection on statistics for biologists contains articles on many of the points above.

Software and code

Policy information about availability of computer code

Data collection	Orbitrap Eclipse Tribrid MS (Thermo Scientific) with Xcalibur Ver 4.3.73.11<br>Q Exactive Plus (Thermo Fisher) with Xcalibur Ver 4.0.27.19<br>Zeiss LSM 710 with Zen 2.1 SP2 version 130.2.518
Data analysis	MaxQuant (Ver. 1.6.14.0)<br>Perseus (Ver 1.6.14.0)<br>String-DB (Ver. 11.0b, https://version-11-0b.string-db.org/)<br>SCPCompanion (ver. 15.0, https://github.com/scp-ms/SCPCompanion)<br>Microsoft office 365<br>GraphPad Prism Ver 8.3.0

For manuscripts utilizing custom algorithms or software that are central to the research but not yet described in published literature, software must be made available to editors and reviewers. We strongly encourage code deposition in a community repository (e.g. GitHub). See the Nature Portfolio guidelines for submitting code & software for further information.

Data

Policy information about availability of data

All manuscripts must include a data availability statement. This statement should provide the following information, where applicable:

- Accession codes, unique identifiers, or web links for publicly available datasets
- A description of any restrictions on data availability
- For clinical datasets or third party data, please ensure that the statement adheres to our policy

Source data for all the figures are provided as Source Data 1 and 2. The mass spectrometry raw data have been deposited to the ProteomeXchange Consortium via the MassIVE partner repository with the dataset identifier MSV000086809 (https://gnps-explorer.ucsd.edu/MSV000086809) (ftp://massive.ucsd.edu/MSV000086809/). Single-cell RNA sequencing data for control RAW cells were downloaded from NCBI Gene Expression Omnibus with accession number GSE94383 (https://www.ncbi.nlm.nih.gov/geo/query/acc.cgi?acc=GSE94383). Single-cell RNA sequencing data for C10 cells were requested from the author and provided in Source Data 3.

Field-specific reporting

Please select the one below that is the best fit for your research. If you are not sure, read the appropriate sections before making your selection.

[x] Life sciences [ ] Behavioural & social sciences [ ] Ecological, evolutionary & environmental sciences

For a reference copy of the document with all sections, see nature.com/documents/nr-reporting-summary-flat.pdf

Life sciences study design

All studies must disclose on these points even when the disclosure is negative.

Sample size	No statistical methods were used to predetermine sample size in all experiments. In case of the experiment for single-cell level peptides of three cell lines, sample size were determined based on published sample size usages for similar experiments (PMID:32127492; PMID:32639140)
Data exclusions	In figure 5a, four data were excluded based on the criteria of Pearson's correlation among samples. (Two C10, one RAW, and one SVEC single-cell data)
Replication	Experiments were performed multiple times, and most experiments were reproduced at least three times with similar results.
Randomization	Not relevant to the study, as only cultured cells were used for technology evaluation.
Blinding	Not relevant to the study. We used well known cultured cells for technology evaluation.

Reporting for specific materials, systems and methods

We require information from authors about some types of materials, experimental systems and methods used in many studies. Here, indicate whether each material, system or method listed is relevant to your study. If you are not sure if a list item applies to your research, read the appropriate section before selecting a response.

Materials & experimental systems

n/a	Involved in the study	
[ ]	[x]	Antibodies
[ ]	[x]	Eukaryotic cell lines
[x]	[ ]	Palaeontology and archaeology
[x]	[ ]	Animals and other organisms
[x]	[ ]	Human research participants
[x]	[ ]	Clinical data
[x]	[ ]	Dual use research of concern

Methods

n/a	Involved in the study	
[x]	[ ]	ChIP-seq
[x]	[ ]	Flow cytometry
[x]	[ ]	MRI-based neuroimaging

Antibodies

Antibodies used	Three recombinant antibodies were purchased from Abcam (Cambridge, MA, USA); Anti-NCAM1 (ab220360, 1:1000 dilution), Anti-CD14 (ab221678, 1:1000 dilution), and Anti-BST2 (ab246508, 1:2000 dilution). Alexa Flour 546 goat anti-rabbit IgG (Invitrogen, Cat#A11010, 1:1000 dilution) was used as a secondary antibody.
Validation	For all antibodies, validation was performed by the manufacturers using Western blot, Immunofluorescence, Immunocytochemistry, or Immunohistochemistry. Published paper using the antibody are also included in webpage. See https://www.abcam.com/ncam1-

antibody-epr21827-ab220360.html; https://www.abcam.com/cd14-antibody-epr21847-ab221678.html; https://www.abcam.com/bst2tetherin-antibody-epr23597-202-ab246508.html

Eukaryotic cell lines

Policy information about cell lines

Cell line source(s)	Three murine cell lines (RAW 264.7, a macrophage cell line; C10, a respiratory epithelial cell line; SVEC, an endothelial cell line) were obtained from ATCC. Three leukemia cell lines (MOLM-14, K562, and CMK) were kindly provided by Dr. Anupriya Agarwal at Oregon Health & Science University. The MOLM-14 cells were originally established from the peripheral blood of a patient at relapse of acute monocytic leukemia by Dr. Matsuo et al. at Fujisaki Cell Center in Japan. K562 cells were obtained from ATCC. CMK cells were obtained from the German National Resource Center for Biological Material.
Authentication	None of the cell lines used were authenticated, because these cells were used as model samples for technology evaluation
Mycoplasma contamination	The cell lines were not tested for mycoplasma contamination
Commonly misidentified lines (See ICLAC register)	No misidentified cell lines were used in this study
